# METCAM/MUC18 Decreases the Malignant Propensity of Human Ovarian Carcinoma Cells

**DOI:** 10.3390/ijms19102976

**Published:** 2018-09-29

**Authors:** Guang-Jer Wu

**Affiliations:** 1 Department of Bioscience Technology and Center for Biomedical Technology, Chung Yuan Christian University, Chung Li 32023, Taiwan; guangj.wu@gmail.com; Tel.: +886-3-265-3507 or 3508; Fax: +886-3-265-3599; 2 Department of Microbiology & Immunology and Winship Cancer Institute, Emory University School of Medicine, Atlanta, GA 30322, USA

**Keywords:** METCAM/MUC18, human ovarian carcinoma cells, SK-OV-3 & BG-1 cell lines, EMT, *SC* & *IP* injections, tumorigenicity, ascites formation, mechanisms, female athymic nude mice

## Abstract

METCAM/MUC18 is an integral membrane cell adhesion molecule (CAM) in the Ig-like gene super-family. It can carry out common functions of CAMs which is to perform intercellular interactions and interaction of cell with extracellular matrix in tumor microenvironment, to interact with various signaling pathways and to regulate general behaviors of cells. We and other two groups previously suggested that METCAM/MUC18 probably be utilized as a biomarker for predicting the malignant tendency of clinical ovarian carcinomas, since METAM/MUC18 expression appears to associate with the carcinoma at advanced stages. It has been further postulated to promote the malignant tendency of the carcinoma. However, our recent research results appear to support the conclusion that the above positive correlation is fortuitous; actually METCAM/MUC18 acts as a tumor and metastasis suppressor for the ovarian carcinoma cells. We also suggest possible mechanisms in the METCAM/MUC18-mediated early tumor development and metastasis of ovarian carcinoma. Moreover, we propose to employ recombinant METCAM/MUC18 proteins and other derived products as therapeutic agents to treat the ovarian cancer patients by decreasing the malignant potential of ovarian carcinoma.

## 1. Introduction-Present Status of Ovarian Carcinoma and The Importance of Cell Adhesion Molecules in the Malignant Progression of Carcinoma in General

Ovarian carcinoma is the fifth leading cause of female cancer death in USA [1]. The reason for its high lethality is that most early disease is asymptomatic and the cancer stays undiagnosed until it is too late (at advanced stages), at then the carcinoma has spread through the peritoneal cavity [2]. Early ovarian carcinoma may be effectively treated with a high survival rate. However, one of the major problems is the lack of a good biomarker for detecting the early disease. This is because the validated marker for ovarian cancer, CD125, is not a diagnostic or prognostic marker even it is present in the serum of more than 80% of women with ovarian carcinoma [3]. Furthermore, an efficient therapy for the disease at advanced stages is not available since the recurrent cancer is highly resistant to chemotherapy. Major problems for treating ovarian carcinoma include: (a) the carcinoma is heterogeneous at both histological and molecular levels, manifesting more than four major subtypes (serous adenocarcinoma (40%), endometrioid adenocarcinoma (20%), mucinous adenocarcinoma (10%) and clear cell carcinomas (5%)) [4,5]; (b) dependable and specific biomarkers for an accurate diagnosis of each subtype are absent [2]; and (c) the detailed knowledge of the emergence of ovarian carcinoma and how it progresses to malignant form remain elusive ([6] for a review). Thus, a new diagnostic marker is still needed to detect the early disease. It is also highly desirable if a new therapeutic strategy can be designed from a more comprehension of the detailed processes in the malignant progression of the carcinoma.

Cell adhesion molecules (CAMs) very likely play a substantial role in the malignant progression of carcinomas, since they govern the social behaviors, influence outlasting, proliferation and growth of tumor cells and modulate generation of new blood vessels in the tumor microenvironment [7]. We have focused our research on the possible METCAM/MUC18 expression in normal and cancerous ovarian [8] and its effects on the development of the carcinoma. From the results, as described in the following sections, we believe that METCAM/MUC18 may not be a useful marker for early diagnosis of the carcinoma but it certainly is useful for reducing the malignant tendency of ovarian carcinoma.

In this review, we show negative correlation of the level of METCAM/MUC18 expression in various human ovarian carcinoma cell lines with their malignant status. We indicate negative effects of METCAM/MUC18 over-expression on the epithelial-to-mesenchymal transition and on the tumorigenesis and metastasis of two human ovarian carcinoma cell lines. Then we propose preliminary detailed knowledge of how METCAM/MUC18 may induce suppression of the malignant tendency of human ovarian carcinoma cell lines. Finally, we describe perspectives of the studies and suggest possible clinical applications.

## 2. Cell Adhesion Molecules Involved in Regulating the Malignant Potential of Ovarian Carcinoma

CAMs participate in many significant normal biological functions, such as organ generation, tissue organization, de novo formation of endothelial cells from mesoderm cell precursors (vascularization) and formation of new blood vessels from pre-existing ones (angiogenesis), immune response, inflammation, wound healing and cellular general behaviors [7]. An altered expression of CAMs can impact tumorigenesis, because CAMs control general behaviors of cells by impacting the adhesion status of cells and cross-interacting with intracellular signal transduction pathways [7]. Aberrant expression of CAMs also impacts distant organ-dissemination of carcinoma cells, because CAMs orchestrate complex interactions of tumor cells with various stromal cells in the tumor microenvironment, resulting in augmentation or reduction of the spreading potential of carcinoma cells [7,8,9]. In the past several decades, we have focused our research activities on investigating the possible role played by METCAM/MUC18 in impacting the malignant tendency of several carcinomas, such as breast carcinoma, melanoma, nasopharyngeal carcinoma and prostate cancer [9]. We found that METCAM/MUC18 might be able to promote or suppress tumorigenesis and metastasis of these cancers [9].

Altered expression of several CAMs, such as mucins [3,10], integrins [11], CD44 [12], L1CAM [13], cadherin [14], claudins [15], EpCAM [16], ALCAM [17] and METCAM/MUC18 [8,18,19], is linked to the malignant progression of ovarian carcinoma. Some of them, such as MUC4 [10], CD44 [12], L1CAM [13], ALCAM [17] and P-cadherin [20], promote the malignant tendency of ovarian carcinoma cells. However, some CAMs, such as β3-integrin [21], E-cadherin [14], claudin-3, 4, &7 [15], EpCAM [16] and KAI1 [22], suppress the malignant progression of the cancer. Thus, cell adhesion molecules also perform central functions in the malignant tendency of ovarian carcinoma. Recently we also investigated the possible role played by METCAM/MUC18 in impacting the malignant tendency of epithelial ovarian tumors [8,23,24].

## 3. A Dual Role Played by METCAM/MUC18 in Impacting the Malignant Tendency of Several Carcinomas

**Figure 1:**
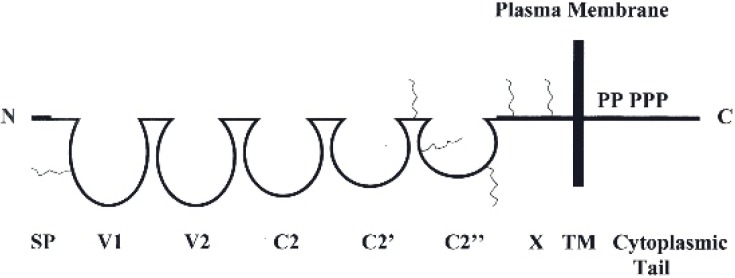
Human METCAM/MUC18 protein structure. SP represents the signal peptide sequence, V1, V2, C2, C2’, C2’’ as the five Ig-like domains (each held by a disulfide bond) and X for one domain (without any disulfide bond) in the extracellular region and TM for transmembrane domain. P represents five potential phosphorylation sites in the cytoplasmic tail. The six conserved N-glycosylation sites are indicated by wavy lines in the extracellular domains of V1, the interdomain between C2’ and C2”, C2’’ and X.

METCAM/MUC18 was first discovered to be profoundly expressed on the cellular surface of the majority of malignant human melanomas (thus named as MUC18) and suggested to play a central role in the progression of human melanoma (thus was originally named as MCAM and Mel-CAM) [25]. Later, however METCAM/MUC18 was not found to be exclusively expressed in melanoma but also expressed in other epithelial tumors [9,26]. Furthermore, it did not initiate the transformation of normal cutaneous melanocytes to melanoma [26]. METCAM/MUC18 also bears other alternative names, such as S-endo1, CD146, or A32 [9,25]. METCAM/MUC18 is a cell adhesion molecule (CAM) in the Ig-like gene superfamily and also a component of the cellular membrane. The naked human METCAM/MUC18 has a total of 646 amino acids, which includes 558 amino acids as the N-terminal extra-cellular domain, 24 amino acids as the transmembrane domain and 64 amino acids of a short intra-cellular cytoplasmic domain at the C-terminal, as shown in the following Figure 1 [9,25].

Figure 1 shows the N-terminal extra-cellular domain of the protein, which is composed of a signal peptide sequence (SP) and five immunoglobulin-like domains and one X domain [9,25]. The intracellular cytoplasmic domain has one, three and one protein kinase consent sequences potentially to be phosphorylated by PKA, PKC and CK2, respectively [9,25]. In addition, there are eight possible N-glycosylation sites, of which six are conserved between human and mouse proteins, in the extracellular domain. METCAM/MUC18 is conserved in mouse, in which the amino acid sequences of mouse METCAM/MUC18 are 72.6% identical to the human METCAM/MUC18 [26]. Therefore, both human and mouse METCAM/MUC18’s can similarly perform common CAMs functions, such as controlling general cell behaviors by modulating cell signaling and impacting the adhesion status of cells. Furthermore, over-expression of both human and mouse METCAM/MUC18’s similarly impacted tumor cells in in vitro motility and invasiveness, in vitro and in vivo tumorigenesis and in vivo metastasis [9,26].

Human METCAM/MUC18 is expressed in about ten normal tissues: hair follicular cells, smooth muscle cells, endothelial cells, cerebellum, basal cells of the lung, activated T cells, intermediate trophoblasts [27], breast epithelium [28,29], ovarian epithelium [8] and nasopharyngeal epithelium [30]. The protein is also expressed in a handful of carcinomas, such as melanoma, prostate adenocarcinoma, osteosarcoma, breast carcinoma and intermediate trophoblast tumors [9,27]. Our studies also indicate that over-expression of METCAM/MUC18 augments tumorigenesis of prostate adenocarcinoma [31], breast carcinoma [28,29] and nasopharyngeal carcinoma type II [32,33] but it does not have an effect on tumorigenesis of melanoma [26,34]. METCAM/MUC18 over-expression also perpetuates the distant organ-dissemination of prostate cancer [31] and augments the distant organ-dissemination of melanoma [26,34] and breast carcinoma [28,29].

In contrast, over-expression of METCAM/MUC18 decreases tumorigenesis of a mouse melanoma cell line, K1735-9 [26,35], nasopharyngeal carcinoma type I [32,33] and perhaps hemangiomas [9]. METCAM/MUC18 over-expression also decreases the distant organ-dissemination of the mouse melanoma cell line, K1735-9 [26,35].

The impact of METCAM/MUC18 over-expression on malignant tendency of ovarian carcinoma has not been systematically investigated. The recent findings in this aspect are outlined in the following section.

## 4. METCAM/MUC18 Acts a Negative Role in Malignant Propensity of Ovarian Carcinoma

METCAM/MUC18 overexpression was first observed to be significantly correlated with the ovarian carcinomas at advanced stage and with the serous and undifferentiated subtypes of the tumors. Since METCAM/MUC18 is expressed at a higher level in the carcinoma than residual disease, it was suggested to be useful for prognosticating tumor relapse and as a self-reliant predicting marker for poor prognosis of ovarian carcinoma [18]. The above notion was also consistent with our findings that METCAM/MUC18 expression appears to be linked with the pathological stages of ovarian carcinoma [8]. Similar to our findings, another group [19] also reported that METCAM/MUC18 expression is higher in metastatic lesions of ovarian carcinoma than other types of pathological ovarian carcinomas [8]. They further showed that using siRNAs to decrease the intrinsic METCAM/MUC18 expression in the SK-OV-3 cell line increases apoptosis and reduces cell spreading and invasion in vitro [19]. Taken together, the previous studies from three independent groups appear to implicate that METCAM/MUC18 promotes the malignant propensity of ovarian carcinoma cells. But this hypothesis has not been confirmed by animal studies. To scrutinize this notion, we studied the impact of METCAM/MUC18 over-expression on in vitro social behaviors and tumorigenesis and on in vivo malignant propensity of human ovarian carcinoma SK-OV-3 and BG-1 cells in an athymic nude mouse model [23,24]. The results are presented in the following sections.

### 4.1. Comparing the Level of METCAM/MUC18 Expression in Ovarian Cancer Cell Lines Established from Malignant Ascites and from Adenocarcinomas

To investigate if the above hypothesis is correct, we compared the expression levels of METCAM/MUC18 in one immortalized normal human ovarian epithelial cell line (IOSE) and five human ovarian cancer cell lines, which were established from primary adenocarcinoma or metastatic ascites [23]. If we assumed that the expression level of METCAM/MUC18 in a positive control, human melanoma cell line SK-Mel-28, was 100%, the expression level of the protein in the IOSE cell line was about 10% and the expression level of the protein in five ovarian cancer cell lines was ranged from zero to 50%. We further noticed that the level of the protein expressed in two (HEY and CAOV3) out of three cell lines established from primary adenocarcinomas was 31–50%; however, it was 1–11% in the two cell lines established from malignant ascites (SKOV3 and NIHOVCAR3). Thus, the expression level of the protein in the cell lines from malignant ascites was weaker than those from primary adenocarcinomas. From this result, it may imply that METCAM/MUC18 may not promote the malignant propensity of ovarian carcinoma, which is opposite to the above notion concluded from correlation. However, this notion must be further scrutinized with in vitro and in vivo tests. For this purpose, the above information also allowed us to take advantage of the two ovarian carcinoma cell lines, which much weakly expressed METCAM/MUC18. BG-1, which was established from a poorly differentiated adenocarcinoma), did not expressed any METCAM/MUC18. SK-OV-3, which was established from an adenocarcinoma metastasis as malignant ascites, expressed only 1% of the protein.

### 4.2. Enforced Expression of METCAM/MUC18 in the SK-OV-3 and BG-1 Cell Lines

To investigate if METCAM/MUC18 expression alters the in vitro and in vivo behaviors of the ovarian cancer cells, it should be useful to enforcedly increase the expression of the gene in the two cell lines, SK-OV-3 and BG-1, which weakly expressed METCAM/MUC18. For this purpose, we transfected the human METCAM/MUC18 cDNA, which was inserted in a mammalian cells-expressible vector, pcDNA3.1+, into these cell lines and selected for the high expressing G418^R^ clones [23,24]. Then we used a few high-expressing clones and a vector control clone for investigating the impact of METCAM/MUC18 over-expression on in vitro cellular motility and invasiveness and on in vivo tumor and ascites formation in mouse models.

### 4.3. METCAM/MUC18 Over-Expression Decreased Epithelial-to-Mesenchymal Transition (EMT) of SK-OV-3 and BG-1 Cells

Activation of the epithelial-to-mesenchymal transition (EMT) is critical for the acquisition of the malignant nature of carcinoma cells ([36] for a review]). EMT is a process by which the carcinoma cells with an initial characteristic of polarized stationary epithelial cells undergo multiple biochemical changes and obtain a characteristic of motile and spindle-shaped mesenchymal cells. Thereafter, the cancer cells undergo disruption of cell-cell adherence and cell-extracellular matrix, migrate out of primary sites and invade through the basement membrane and then disseminate to distant organs via circulatory systems. In distant organs, they may remain mesenchymal-like or re-differentiate back to epithelial cells via a process recognized as mesenchymal-to-epithelial transition (MET). Therefore, EMT may be a process prerequisite to distant organ-dissemination. In addition to increased motility and invasiveness, carcinoma cells by way of EMT may become stemness, guarded from aging, apoptosis and immune surveillance, and insensitive to any types of therapy [36]. The extent of EMT in cells usually can be estimated by the degree of in vitro motility and invasiveness of the cells. The above G418^R^ clones were subjected to the determination of the effects of enforced expression of METCAM/MUC18 on in vitro motility and invasiveness.

From the studies of the impacts of over-expression of METCAM/MUC18 on in vitro motility and invasiveness of the high-expressing clones of SK-OV-3 and BG-1 cell lines, we observed that METCAM/MUC18 over-expression decreased the in vitro motility and invasiveness of the clones derived from both cell lines. These results strongly suggest that METCAM/MUC18 overexpression reduces the EMT ability of SK-OV-3 and BG-1 cells and METCAM/MUC18 expression directly decreases the EMT of these cells [23,24]. However, precaution must be taken that not all tumor cell lines manifest EMT in vitro [36].

### 4.4. METCAM/MUC18 Over-Expression Decreased In Vivo Tumorigenesis and the Malignant Propensity of the Human Ovarian Carcinoma Cell Line SK-OV-3

To examine the above hypothesis that METCAM/MUC18 may promote the malignant propensity of ovarian carcinoma [8], we used the above clones to test the impact of METCAM/MUC18 over-expression on tumorigenesis and malignant propensity of the cells in an athymic female nude mouse model via two injection routes, the (non-orthotopic) subcutaneous (SC) injection route and the (orthotopic) intraperitoneal cavity route [23].

After *SC* injection of the clones/cells, we found that tumor proliferation and the final tumor weights of the METCAM/MUC18-expressing clones were much less than that of the control (vector) clone, indicating that METCAM/MUC18 over-expression reduced the tumorigenesis of SK-OV-3 cells in the nude mouse model [23]. We deducted that METCAM/MUC18 over-expression suppressed in vivo tumorigenesis of SK-OV-3 cells at non-orthotopic (ventral and dorsal) subcutaneous sites. Additionally, the tumors caused by METCAM/MUC18-expressing clone were only restricted to small areas, as shown in the results of histology and immunohistochemistry (IHC) [23], whereas the tumors caused by the control (vector) clone became frank tumors, further suggesting that tumors from the METCAM/MUC18-expressing clone appeared to be dormant. Hence, METCAM/MUC18 appears to act in similarity to other tumor/metastasis suppressors in other tumor cells [37].

Then, we further investigated effects of METCAM/MUC18 over-expression on tumorigenesis of SK-OV-3 cells after injecting the above clones at the orthotopic sites (in the intraperitoneal (*IP*) cavity). We found that the mice injected with cells from the control vector clone formed swollen abdomen, whereas the mice injected with the METCAM/MUC18-expressing clone did not develop swollen abdomen. After surgically opening the abdominal cavities, we found that the final weights of abdominal tumors and volumes of ascites in the group injected with the control vector clone were significantly heavier and larger than those injected with the METCAM/MUC18-expressing clone. We deduced that over-expression of METCAM/MUC18 decreased the tumorigenicity and ascites formation of SK-OV-3 cells at orthotopic (*IP* cavities) site in nude mice [23].

From the above results, METCAM/MUC18 expression in SK-OV-3 cells suppressed tumor proliferation and growth of the cells at both the non-orthotopic *SC* sites and the orthotopic *IP* site. Thus, we have provided evidence to conclusively prove that METCAM/MUC18 is a new tumor and metastasis suppressor for the malignant propensity of human ovarian carcinoma cells [23]. However, precaution must be taken that the tumor suppressor effects of METCAM/MUC18 may not be manifested in other human ovarian carcinoma cell lines when the non-orthotopic route of injection is used, as shown in the case of BG-1 cell line below.

### 4.5. METCAM/MUC18 Over-Expression Decreased In Vitro and In Vivo Tumorigenesis of the Human Ovarian Carcinoma Cell Line BG-1

Anchorage-independent colony formation in soft agar has been used as an in vitro method to determine the tumorigenicity of most cancer cells, as an alternative method to determine the tumorigenicity in model animals (in vivo tumorigenicity) [38]. The in vitro tumor formation ability of a METCAM/MUC18-high expressing BG-1 clone was reduced 5.5-fold in comparison to that of the empty vector control clone, suggesting that enforced expression of METCAM/MUC18 in BG-1 cells repressed in vitro tumor formation [24]. However, this in vitro method as an alternative to determine the tumorigenesis in animal models may not be applicable to all tumor cell lines [38]. This notion was further scrutinized by the in vivo tumorigenesis tests, as described next

We further carried our studies on impacts of METCAM/MUC18 expression on tumorigenesis of BG-1 cells in an athymic nude mouse model also via two injection routes, the (non-orthotopic) subcutaneous (SC) injection route and the (orthotopic) intraperitoneal cavity route [24]. After *SC* injection of the two METCAM/MUC18-expressing clones/cells and one empty vector control clone, the final tumor weights of the METCAM/MUC18-expressing clones and the empty vector control clone were found to be not statistically different, indicating that increased METCAM/MUC18 expression minimally impacted the final tumor weights via this injection route. We concluded that METCAM/MUC18 over-expression did not significantly induce in vivo tumorigenesis when non-orthotopic site was injected with the cells [24].

In contrast, when the above clones were intraperitoneally injected in nude mice, the final tumor weight of the METCAM/MUC18-expressing BG-1 clone was significantly decreased in comparison to that of the empty vector control clone, indicating that increased METCAM/MUC18 expression significantly decreased in vivo tumorigenesis of BG-1 cells [24]. Taken together, METCAM/MUC18 over-expression reduced in vivo tumorigenesis via the intraperitoneal injection route. However, no ascites was found in the abdominal cavity either from injecting the METCAM/MUC18-expressing clones or the empty vector control clone.

Taken together, a higher expression level of METCAM/MUC18 significantly reduced the in vitro tumor formation and the in vivo tumor proliferation at the orthotopic site. Thus, in addition to SK-OV-3 cells [23], over-expression of METCAM/MUC18 also suppresses the malignant propensity of human ovarian carcinoma BG-1 cells, Wu, G.-J. [24] suggesting that this conclusion is generally applicable to human ovarian carcinoma cells. Surprisingly, BG-1 clones/cells were not as tumorigenic as SK-OV-3 cells in the nude mouse model, in addition to no ascites formation in the abdominal cavity. We did not know the reason for this difference between the two cell lines. One possible reason may be that due to the malignancy of the BG-1cell line, which was established from a poorly differentiated adenocarcinoma [24], is not as advanced as the SK-OV-3 cell line, which was established from malignant ascites metastasized from an adenocarcinoma [23]; thus BG-1 cells may require additionally altered physiological conditions to manifest the effect of huMETCAM/MUC18 (human METCAM/MUC18). One possible physiological condition may be that the BG-1 cell line requires estrogen for augmentation of the tumorigenicity, since it contains estrogen and progesterone receptors [24]. Other altered physiological factors are not ruled out and a systematic investigation may be required [24].

As shown above, precaution must be taken that the biological effects demonstrated in one cancer cell line may not be completely reproduced in other cancer cell line, because the origin from which each cell line was established is different, as each tumor or metastatic lesion is a mixed bag of tumor/cancer cells with slightly different mutated genotypes.

### 4.6. Possible Mechanisms of the METCAM/MUC18-Suppressed Malignant Propensity of SK-OV-3 Cells

To understand further the detailed knowledge of METCAM/MUC18-mediated suppression of the malignant propensity of ovarian carcinoma cells, perhaps some clues may be deduced from what we know from the METCAM/MCU18-mediated tumorigenesis of various tumor cell lines, such as cancers of breast and prostate, melanoma and nasopharyngeal carcinoma. METCAM/MUC18 could impact many downstream signaling pathways that regulate proliferation, survival pathway, apoptosis, metabolism and angiogenesis of various tumor cells [7,9]. For this purpose, we carried out preliminary studies by using Western blot analyses to examine whether METCAM/MUC18-mediated suppression indeed also impacted expression of similar downstream effectors, such as indexes of apoptosis/anti-apoptosis, proliferation, survival, aerobic glycolysis and angiogenesis [23]. From the results of measuring the expression levels of Bcl2, Bax, PCNA, LDH-A, pan-AKT, phospho-AKT (Ser 473) and the ratio of phospho-AKT/AKT in the lysates of the tumors induced by SK-OV-3 clones, we conclude that overexpression of METCAM/MUC18 may decrease in vivo tumorigenesis and malignant propensity of ovarian carcinoma cells by reducing the absolute levels of pan-AKT and phospho-AKT, which in turn decreases proliferation, aerobic glycolysis and angiogenesis but not by altering apoptosis/anti-apoptosis and survival pathways [23]. This conclusion agrees to findings in clinical specimens [8]. However, precaution must be taken that the true mechanism may require a systematic investigation of the key members in each pathway.

## 5. Conclusions

**Figure 2:**
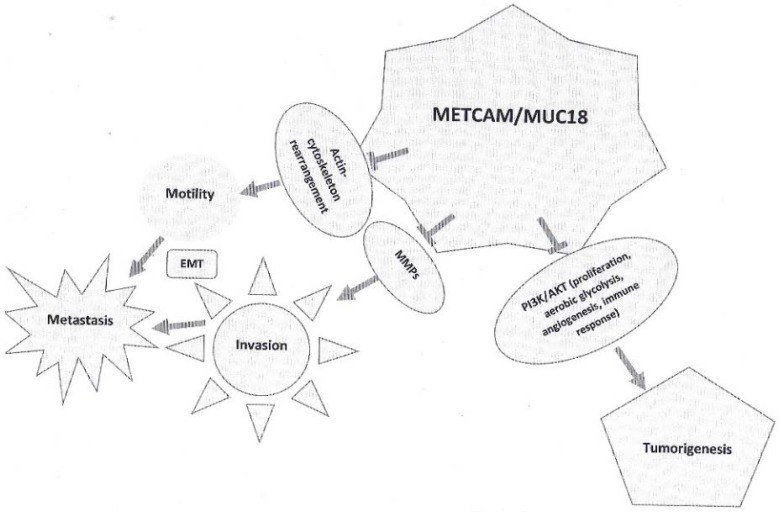
The key roles of METCAM/MUC18 in the tumorigenesis and metastasis of ovarian carcinoma cells.

We offered solid evidence to strongly suggest that METCAM/MUC18 is a new suppressor for the tumorigenesis and malignant propensity of the two human ovarian carcinoma cell lines, SK-OV-3 and BG-1 [23,24]. (a) METCAM/MUC18 was expressed in malignant cell lines at a lower level than in primary adenocarcinomas, suggesting that METCAM/MUC18 may down regulate the malignant propensity of ovarian carcinoma. (b) METCAM/MUC18 over-expression reduces EMT of SKOV3 and BG-1 clones/cells. (c) METCAM/MUC18 expression inhibited the subcutaneous tumorigenicity and tumorigenicity and ascites formation of SK-OV-3 clones/cells in the intra-peritoneal cavity of an athymic nude mouse model. METCAM/MUC18 expression also inhibited in vitro tumorigenicity and in vivo tumorigenicity of BG-1 clones/cells. All these manifested processes were not due to the modification of the protein in the clones after being injected into the animal model, since the molecular weights of METCAM/MUC18 expressed in the tumors and ascites cells were identical to that in the injected clones/cells. From preliminary mechanical studies, we suggest that METCAM/MUC18 may subdue in vivo tumorigenesis and malignant progression of ovarian carcinoma cells by reducing their ability in proliferation, aerobic glycolysis (metabolism) and angiogenesis perhaps via suppressing the PI3K-AKT signaling pathway. The key roles of METCAM/MUC18 in suppression of human ovarian carcinoma cell lines are summarized and illustrated in Figure 2, as shown below.

## 6. Discussion

The first novel aspect of the above conclusion is that it appears to disagree with an apparently positive correlation of clinical prognosis with the increased expression of METCAM/MUC18 in malignant ovarian carcinoma specimens [8,18,19]. We suggest that the positive correlation in this case is fortuitous and it should not be used to assume a positive role of METCAM/MUC18 in the progression of ovarian carcinoma without the scrutiny in an animal model.

The second novel aspect of the above conclusion is that the tumor suppressor role of METCAM/MUC18 in the human ovarian carcinoma cell lines [23,24] is also opposite to the previously established role of METCAM/MUC18 in other cancer cells in that it acts as a tumor promoter in both prostate cancer cells and breast cancer cells and as a metastasis promoter in human melanoma cells, prostate cancer and breast cancer [26,28,29,30,31,34]. The suppressor role of METCAM/MCU18 in the malignant progression of human ovarian carcinoma cells has also been demonstrated in other carcinoma cells, such as a mouse melanoma cell line, K1735-9 [35] and one NPC cell line, NPC-TW01 [32,33].

The third novel aspect of the findings is that METCAM/MUC18 seems to act a dual role, either as a promoter or suppressor, in the malignant propensity of several tumor cell lines [9]. It either plays an opposite role in different cancer types or in different clones/sublines of the same cancer type; however, it does not act a dual role in the same clones/sublines of the same cancer type [9]. Therefore, we suggest that the dual role of METCAM/MUC18 behaving in the malignant propensity of different carcinomas is possibly due to the consequence of interaction(s) of METCAM/MUC18 with different intrinsic factors, which modulate its functions in different tumor clones/cells or types. One of these factors may be METCAM/MUC18’s heterophilic ligands, which, however, have not been found [9]. Interaction of METCAM/MUC18 with different intrinsic partners may end up either increasing or decreasing aerobic glycolysis, proliferation, angiogenesis, other growth-promoting pathways and also changing tumor cell motility, invasiveness and vascular metastasis, which eventually leads to either promotion or suppression of tumorigenesis and distant organ-dissemination. The dual behavior of METCAM/MUC18 in the malignant propensity of human carcinomas is not an unusual surprise, since many biological molecules have recently also been revealed to act a dual role in the progression of cancer. The three most famous examples are: TGF-β, which is context dependent, since it behaves as a tumor suppressor in the early stage of tumorigenesis but as a progression promoter in the late stage [7], VEGF, which acts a dual role in tumor progression dependent upon the levels of its expression and the context and timing of its modulation [39] and c-myc, which is modulated by different partners to act a dual role in tumor progression [40].

The fourth novel aspect is that a tumor and metastasis suppressor role played by human METCAM/MUC18 in the malignant propensity of human ovarian carcinoma cells and other cancer cell lines appears to suggest the probability that METCAM/MUC18 may trigger on tumor dormancy [37], which is intriguing for future research. Does it inhibit the intrinsic growth, suppress immunological response, and/or reduce angiogenesis of those cancer cells [41]? The answer requires many years of further studies.

### 6.1. Perspectives and Clinical Applications

#### 6.1.1. Perspective

The current studies have laid an important biological basis for inspiring future intense investigation to further understand the detailed knowledge of METCAM/MUC18-mediated suppression of ovarian tumorigenesis and metastasis. However, the current research has not completely proven that the suppressor role of METCAM/MUC18 is generally applicable to all human ovarian cell lines, such as HEY, CAOV-3 and NIHOVCAR3 and other cell lines. To prove that the suppressor role of METCAM/MUC18 in all human ovarian carcinoma cell lines, human ovarian carcinoma cell lines other than the two used in the above research should also be tested by using siRNA to knock down endogenously expressed METCAM/MUC18. To further understand the role of this CAM in regulating cancer metastasis, other future experiments may include: (a) searching for the heterophilic ligands or partners of METCAM/MUC18 [9], (b) systematic investigation of the three mechanisms in the tumor and metastasis dormancy (for examples, what are the key members involved in intrinsic growth inhibition, immunological suppression, and/or reducing angiogenesis?) [41], (c) possible miRNAs [42] and long non-coding RNAs [43] upstream and downstream of METCAM/MUC18 involved in the process and (d) the transcriptional control in the processes [9]. Precaution should be taken that a complete picture may only be possibly constructed after all the above studies are successfully executed.

#### 6.1.2. Clinical Applications

Metastasis of primary tumor to distant organs is responsible for 90% cancer-associated mortality. It would be a major success in cancer treatment if it is possible to arrest the metastatic tendency of cancer cells and keep cancer cells confined to primary site. Alternatively, it is also a major success if it is possible to keep the cancer cells at the condition of dormancy, as micro-metastatic lesions. Since metastasis suppressors are able to trigger tumor dormancy, the metastasis suppressor role of METCAM/MUC18 in the malignant propensity of human ovarian carcinoma cell lines may be useful for designing novel therapeutic methods to treat clinical ovarian carcinomas, similar to the clinical application of other metastasis suppressors, such as KISS1, KAI1, nm23, MAP2K4 and some micro-RNAs to arrest other cancers [44]. Three general strategies may be used for this purpose: (a) reconstitution of suppressor genes by gene therapy or activation of the tumor/metastasis suppressor genes by activation of the locus on chromosome, (b) directly administering the recombinant suppressor proteins to the patients, (c) aiming at key members in the downstream pathways that are activated by the loss of metastasis suppressor function. For specific applications of the above strategies, METCAM/MUC18 cDNA gene may be carried by the adenovirus-associated virus vector or a replication-defective adenovirus and used for gene therapy. The endogenous locus (11q23.3) of the METCAM/MUC18 gene on the human chromosome #11 may be transcriptionally activated by treatment with chemical reagents to reverse epigenetic repression [45] or to alter histone modifications to induce chromosome remodeling [46]. The complete or partial METCAM/MUC18 recombinant protein, oligopeptides derived from METCAM/MUC18, or small molecule mimetics of METCAM/MUC18 may also be administered into the ovarian carcinoma patients. In addition, the heterophilic ligand(s) of METCAM/MUC18 may be used as the therapeutic targets. Many key members in the pathways downstream of METCAM/MUC18 could also be put into use as the therapeutic targets. The above strategies may be used in single, or better in combinatory treatment regimen for the patients via withholding ovarian carcinoma cells in a dormant state or detain the disseminating cancer cells at the condition of micro-metastases.

To further design a novel therapeutic strategy to more aggressively stamp out ovarian carcinoma, we may be able to take advantage of both our METCAM/MUC18-derived recombinant proteins, oligopeptides, or small molecule mimetics and the highly effective anticancer platinum drugs to formulate a combinatory treatment strategy [47]. For this purpose, these METCAM/MUC18 derivatives may be covalently linked to a platinum drug (such as carboplatin) to form a new platinum drug [48] and delivered specifically to ovarian carcinoma by properly designed nanoparticles [47,48]. By combining both the tumor/metastasis suppression function of METCAM/MUC18 and the effective cytotoxicity of the platinum drugs this new platinum drug may be used for a more effective treatment of the ovarian cancer patients.
